# Tourists’ attitudes towards ban on smoking in air-conditioned hotel lobbies in Thailand

**DOI:** 10.1136/tc.2009.029686

**Published:** 2009-04-12

**Authors:** V Viriyachaiyo, A Lim

**Affiliations:** 1 Division of Respiratory and Respiratory Critical Care Medicine, Department of Internal Medicine, Faculty of Medicine, Prince of Songkla University, Hat Yai, Thailand; 2 Department of Mathematics and Computer Science, Faculty of Science and Technology, Prince of Songkla Unversity, Pattani Campus, Thailand

## Abstract

**Background:**

Thailand is internationally renowned for its stringent tobacco control measures. In Thailand, a regulation banning smoking in air-conditioned hotel lobbies was issued in late 2006, causing substantial apprehension within the hospitality industry. A survey of tourists’ attitudes toward the ban was conducted.

**Methods:**

A cross-sectional survey of 5550 travellers staying in various hotels in Bangkok, Surat Thani, Phuket, Krabi and Songkhla provinces, October 2005 to December 2006. Travellers aged 15 years or older with a check-in duration of at least one day and willing to complete the questionnaire were requested by hotel staff to fill in the 5-minute questionnaire at check-in or later at their convenience.

**Results:**

Secondhand cigarette smoke was recognised as harmful to health by 89.7% of respondents. 47.8% of travellers were aware of the Thai regulation banning smoking in air-conditioned restaurants. 80.9% of the respondents agreed with the ban, particularly female non-smokers. 38.6% of survey respondents indicated that they would be more likely to visit Thailand again because of the regulation, 53.4% that the regulation would not affect their decision and 7.9% that they would be less likely to visit Thailand again.

**Conclusion:**

Banning smoking in air-conditioned hotel lobbies in Thailand is widely supported by tourists. Enforcement of the regulation is more likely to attract tourists than dissuade them from holidaying in Thailand.

Thailand is internationally renowned for its stringent tobacco control measures. Thailand, with more than 30 years’ experience of tobacco control campaigning (among the first 40 countries to ratify the Framework Convention on Tobacco Control (FCTC) needed to bring the treaty into force), issued a regulation in August 2006 advising that smoking would be banned in air-conditioned hotel lobbies. The regulation was implemented from 23 November 2006. Rumours of the intended regulation raised substantial apprehension in the hospitality industry that the regulation might negatively impact the national economy. Tourism generated 30% of total national revenue in 2006^1^ and sections of the hospitality industry argued that the ban would dissuade a significant number of tourists from returning to Thailand. To assist government decision-making, a survey of tourists’ opinions in Thailand regarding the regulation was therefore conducted before the announcement of the intended regulation to examine the link, if any, between the enforcement of the ban and a decline in tourism.

## METHODS

A cross-sectional survey of tourists staying at 3-star and 4-star hotels in Bangkok, Surat Thani, Phuket, Krabi and Songkhla provinces, the most popular tourism sites in Thailand, was conducted between October 2005 and December 2006. Almost all of the hotel lobbies in Bangkok and Songkhla are air-conditioned, whereas in Phuket, Krabi and Surat Thani most hotel lobbies are open air. Hotel executives and staff agreed to participate in conducting the survey. Travellers aged 15 years or older with a check-in duration of at least one day, willing to complete the questionnaire in one of six language versions including Thai, English, Japanese, Korean, Chinese and German were requested by the hotel staff to fill in the 5-minute questionnaire at check-in or later at their convenience. The questionnaire form was piloted to tourists who stayed at a hotel in Songkhla and Krabi province with subsequent readjustment accordingly to be consistent with the purpose of the study. Of the 5567 questionnaires returned, 17 were excluded because of incomplete or unclear information recorded.

## RESULTS

Most guests surveyed were middle-aged adults (average age 36.7 (SD 12.9) years) and more than half of the respondents were male (62.4% male, 37.6% female). The locations where the surveys were conducted with the proportions of respondents were: Bangkok (34%), Surat Thani (21%), Phuket (20%), Krabi (13%) and Songkhla province (11%). The travellers were mainly Asians (41%) and white people (38%).The top 10 countries of foreign respondents’ residence were the United Kingdom (12.6%), Australia (11.3%), Singapore (9.6%), Japan (8.6%), Germany (7.5%), the United States (7.1%), Sweden (6.5%), Malaysia (5.6%), the People’s Republic of China (2.7%) and the Republic of Korea (2.5%). In all, 20.8% of the sample were professionals with 3.6% of these working in the healthcare sector, 18% were business entrepreneurs and company/state executives, 17.4% were company employees, 9.6% were students and 6.0% were government officials. Retirees and those with unidentified professions comprised 13% of respondents. Of those who completed the questionnaire, 19.6% identified themselves as former smokers, 32.5% indicated that they were current smokers and 47.9% were non-smokers.

Travellers’ opinions about the regulation are shown in table 1 with group comparisons of various characteristic variables shown in table 2. Secondhand cigarette smoke was recognised as harmful to health by 89.7%. Almost half (47.8%) of the travellers were aware of the Thai regulation banning smoking in air-conditioned restaurants. The majority (80.9%)—especially female non-smokers—agreed with the proposed ban on smoking in hotel lobbies. Moreover, 38.6% indicated that they would be more likely to visit Thailand again if the regulation was introduced, with another 53.4% indicating that the regulation would not affect their decision one way or another, and only 7.9% indicating that they would be less likely to visit Thailand again if there was such a regulation. The groups that would be least likely to visit Thailand because of the regulation were business entrepreneurs, those respondents categorised as either unemployed and of unidentified profession, samples from the Krabi site and travellers from Spain, Hungary, Norway, Austria, South American countries, Israel, Malaysia, Italy and the Republic of Korea and those respondents who were current smokers.

**Table 1:** Opinions of tourists

		No (%)
Secondhand cigarette smoke is harmful		
Agree		4901 (89.7)
Disagree		565 (10.3)
Awareness of Thai regulation banning smoking in air-conditioned restaurants		
Yes		2642 (47.8)
No		2881 (52.1)
Agreement with banning smoking in air-conditioned hotel lobbies		
Yes		4464 (80.9)
No		1054 (19.1)
If regulation was introduced:		
Would be more likely to revisit Thailand		2114 (38.6)
Would be less likely to revisit Thailand		435 (7.9)
Would not affect the decision one way or another		2929 (53.4)

**Table 2:** Variables regarding opinion on returning to Thailand

Variables	No	Percentages			p Value
		More likely to return	Less likely to return	No effect	
Age (SD)	5308	37.3 (13.3)	36.7 (12.1)	36.0 (12.5)	0.001
Sex					
Male	3427	38.6	8.6	52.8	0.029
Female	2066	38.3	6.7	55.0	
Site of collected samples					
Bangkok	1877	42.1	4.8	53.1	<0.001
Surat Thani	1184	29.7	8.3	62.1	
Phuket	1077	32.4	7.1	60.5	
Krabi	721	42.0	15.7	42.3	
Songkhla	619	51.9	9.4	38.8	
Profession					
Professionals	1146	41.3	8.9	49.8	<0.001
Business entrepreneurs/CEO	989	38.8	8.5	52.7	
Company employees PP	957	38.4	6.3	55.4	
Students	535	38.7	5.8	55.5	
Government officials	332	46.1	7.5	46.4	
Unemployed/retired	147	25.9	10.2	64.0	
Media services	126	24.6	5.6	69.8	
Travel agency	91	29.7	8.8	61.5	
Construction workers	81	34.6	7.4	58.0	
Others and unclear	382	40.6	7.3	52.1	
Unidentified	692	36.3	10.0	53.8	

## DISCUSSION AND CONCLUSION

The data showed strong support for the regulation banning smoking in air-conditioned hotel lobbies. This is likely to be due in part to the global trend towards smoke-free indoor areas. Moreover, the leading three tourist source nations (UK, Australia and Singapore) have already banned smoking in hotel lobbies.

Numerous studies have shown that the reduction or elimination of tobacco use will have no negative economic impact for the vast majority of countries, and in some cases will be beneficial.^2^ When individuals do not spend money on tobacco, they spend money on other things, most of which impose far fewer costs on society than tobacco.^3^

The global trend assists in explaining the 90% awareness of the hazards of secondhand smoke and why almost 81% of travellers in the study group (including 21% of former smokers and 25% of current smokers) were in agreement with the proposed regulation banning smoking in air-conditioned hotel lobbies in Thailand. Furthermore, since 2001, announcements have been made on board aeroplanes when approaching Bangkok that smoking is not permitted in the airport. Hence tourists are already given a warning about Thailand’s strict regulations on smoking and it has not deterred tourist arrivals.

Only 8% of travellers (56% being current smokers) claimed to be less likely to return to Thailand if the regulations were implemented. It is possible that introducing the regulation might in fact make Thailand more attractive as a destination. The foreign travellers claiming they were less likely to return were residents of countries with a higher prevalence of smokers among both men (30–65%) and women (17–32%).^4^

The travellers’ surveyed awareness of the regulation preventing smoking in air-conditioned restaurants was quite low, with 52% of the total sample size not knowing about the Thai law. This finding strongly suggests that tobacco control bodies, both in the government and private sectors, need to build more momentum, perhaps in cooperation with the tourism and hospitality industries to raise this awareness.

Tourism has been affected by three exogenous factors since the regulation was introduced: the tsunami disaster hit Thailand during high tourist season in 2007; political unrest closed Suvarnabhumi airport for an extended period in late 2008; and Thailand has, like all nations, been affected by the global recession in 2008–2009. It is therefore not possible to compare tourism rates before and after the regulation and draw any conclusions about the impact of the regulations.

### Study strengths

The large study population of 5550 respondents were from 3-star and 4-star hotels at five favourite tourism sites in Thailand. The foreign travellers were mainly white people and Asians, and are therefore congruent with the distribution of travellers to Thailand researched and published by the Bank of Thailand.^1^

### Study limitations

The survey relied on the willingness of travellers to answer the questionnaires. It is not known how many travellers declined to complete the questionnaire as hotel staff did not keep data on refusals and their diligence in requesting survey completions was not monitored.

**What this paper adds**

Anxieties are sometimes expressed by the hospitality industry that smoke-free laws will alienate tourists and cause them to avoid holiday destinations where smoking is restricted. This survey of over 5000 hotel guests in Thailand showed a large majority of tourists supported a ban on smoking in hotel lobbies. Many of these tourists are from nations where the same restrictions on smoking apply.

## References

[1] Monetary Policy Group (2007). Thailand’s economic and monetary conditions in 2006.

[2] Scollo M Lal A Hyland A Review of the quality of studies on the economic effects of smoke-free policies on the hospitality industry. Tob Control 2003;12:13–20 12612356 10.1136/tc.12.1.13 PMC1759095

[3] World Health Organization 35th Session of the Subcommittee of the Executive Committee on Planning and Programming Washington, DC, 2001

[4] Mackay J Eriksen M The tobacco atlas Myriad Editions Limited, WHO, 2002

